# Sleep patterns among Norwegian nurses between the first and second wave of the COVID-19 pandemic

**DOI:** 10.1186/s12912-021-00628-w

**Published:** 2021-06-21

**Authors:** Siri Waage, Ståle Pallesen, Øystein Vedaa, Hogne Buchvold, Kjersti Marie Blytt, Anette Harris, Bjørn Bjorvatn

**Affiliations:** 1 Norwegian Competence Center for Sleep Disorders, Haukeland University Hospital, Jonas Lies vei 65, 5021 Bergen, Norway; 2 Department of Global Public Health and Primary Care, University of Bergen, Bergen, Norway; 3 Department of Psychosocial Science, University of Bergen, Bergen, Norway; 4 Department of Health Promotion, Norwegian Institute of Public Health, Bergen, Norway; 5 Department of Mental Health, Norwegian University of Science and Technology, Trondheim, Norway; 6 Department of Health and Caring Sciences, Western Norway University of Applied Sciences, Bergen, Norway

**Keywords:** Sleep, COVID-19 pandemic, Nurses

## Abstract

**Background:**

Nurses are in the frontline and play an important role in the battle against the COrona VIrus Disease-2019 (COVID-19) pandemic. Sleep problems among health care workers are likely to increase due to the pandemic. However, it is conceivable that negative health outcomes related to the pandemic fluctuate with the infection rate waves of the pandemic. The present study aimed to investigate sleep patterns among Norwegian nurses, after the first wave, during a period with very low rates of COVID-19.

**Methods:**

Data stemmed from the cohort study “**SU**rvey of **S**hift work, **S**leep and **H**ealth (SUSSH)” among Norwegian nurses. A total of 1532 nurses responded one time to a questionnaire between June and September in 2020 including items about demographics and work, information about COVID-19 and quarantine, sleep patterns and changes in sleep patterns due to the pandemic. Descriptive statistics for all relevant variables were calculated and McNemar tests were used to compare categorical variables.

**Results:**

The majority of nurses (84.2%) reported no change in sleep duration after the first wave of the COVID-19 pandemic compared to before, 11.9% reported less sleep, and 3.9% reported more sleep. Similarly, 82.4% of the nurses reported no change in their sleep quality, whereas 16.2% of the nurses reported poorer sleep quality after the first wave of the pandemic compared to before. The majority of nurses reported no change in their sleep schedule due to the pandemic, although 9.6% of the nurses reported to go to bed later and 9.0% woke up earlier than before the pandemic.

**Conclusions:**

Most existing literature exploring sleep among health care workers during the COVID-19 pandemic has been carried out during periods with high infection rates. In this study we aimed to investigate sleep patterns among Norwegian nurses following the first wave, during a period of low COVID-19 rates in Norway. Most of the nurses reported no change in neither sleep duration, sleep quality, bedtime, nor wake-up times compared to before the pandemic. Still, nearly 12% reported shorter sleep duration, and about 16% reported poorer sleep quality indicating that some nurses experienced worsening of their sleep following the pandemic.

## Introduction

The outbreak of the coronavirus SARS CoV-2 pandemic in 2019 affected societies worldwide and had a major impact on most people’s lives. The implications of the pandemic are not yet fully understood. For some occupational groups the pandemic has implied a profound impact [1]. This concerns in particular health care workers, including nurses, who are in the frontline of the pandemic and play an essential role in the management of the coronavirus and its consequences (i.e., from administering vaccines to treating COVID-19 patients).

Previous studies have shown that health care workers report poor sleep quality under non-pandemic circumstances [2–4], and these difficulties are believed to be exacerbated during the COVID-19 pandemic [5]. Being in the frontline of the COVID-19 pandemic place health care workers at high risk of becoming infected by the virus [6]. Furthermore, studies from previous epidemics such as SARS and Ebola have shown that frontline health care workers carry a significant emotional burden [7]. One meta-analysis published in 2020 focusing on the effects of the COVID-19 pandemic on mental health and sleep among health care workers identified 13 papers of which five included insomnia as an outcome. Four of the five studies were conducted in China during the early phase of the pandemic, and the pooled prevalence of insomnia across the studies was 34.3% [8].

As seen during other virus pandemics, the dynamics of the COVID-19 pandemic have taken the form of periods with high infection rates followed by periods with low infection rates. Thus, in most European countries, including Norway, there were two waves of high infection rates in 2020 – one during the spring and one during the fall [9]. Assumingly, the negative health outcomes related to the pandemic like sleep problems would co-vary consistent with such waves. Most of the published studies on the COVID-19 pandemic focus on how health care workers experience being in the frontline of this worldwide crisis and report overall high numbers of negative health outcomes. For instance, Lin and colleagues [4] examined the subjective sleep status and mental health of health care workers during the peak of the COVID-19 pandemic. Similarly, Wang et al. [2] investigated sleep quality among health care workers in Wuhan which was at the center of the COVID-19 pandemic. These previous studies were conducted in February of 2020. However, it is also important to investigate self-reported health of health care professionals between the peaks of the pandemic waves, as this can provide information about the chronicity of problems caused by the pandemic and also the potential for recovery.

The first laboratory positive COVID-19 case in Norway was confirmed on February 26th, and the first death was reported on March 12th, the day after WHO declared COVID-19 as a pandemic [10]. In Norway, the government introduced nation-wide lockdown to stop the spread of the virus from March 12th, involving strong and invasive measures for the population, encompassing closed schools and kinder gardens, heavy restrictions on sports and cultural activities, social distancing, extensive use of home office at workplaces and closed borders. After the first wave of the pandemic, the Norwegian community was gradually reopened, and from May 11th the schools were opened for all age groups. During the first week of June 2020, the Norwegian Institute of Public Health reported low numbers of COVID-19 cases and deaths among the Norwegian population. In the period of late spring and summer of 2020, several of the most invasive measures were reversed, and in early summer, the daily life started to return to normal, however, still with some restrictions concerning high-risk activities.

It is important to assess possible long lasting sleep problems related to the pandemic. Therefore, the present study aimed to investigate sleep patterns among Norwegian nurses after the first wave of the COVID-19 pandemic, during a period with very low infection rates, and to compare these with sleep patterns before the pandemic hit Norway.

## Methods

### Procedure and participants

Data were collected as a pandemic wave of the ongoing cohort study “**SU**rvey of **S**hift work, **S**leep and **H**ealth (SUSSH)” among Norwegian nurses. The first data collection in this cohort was conducted during winter 2008/2009 (wave 1) when a sample of 5400 nurses was randomly selected from the Norwegian Nurses Organisation’s (NNO) membership roll and invited to participate. The initial sample at this first wave included 2964 nurses. Annual follow-up questionnaires have been sent to all nurses who responded to the first wave, except for nurses who have withdrawn from the study, died or moved to an unknown address. In 2020, during the COVID-19 pandemic, the nurses received a questionnaire with focus on pandemic related variables with the possibility to answer either online (by using SurveyXact at www.sussh.no) or a paper-based version with pre-paid envelopes for returning the completed forms. The questionnaire was sent out the first week of June, with up to two reminders to those who didn’t respond, one after three weeks, and a second reminder in the middle of August. The data collection closed by October 1st. The present study reports findings from this cross-sectional pandemic wave (2020). A total of 1532 nurses responded to the questionnaire (1454 paper version, 78 internet version), yielding a response rate of 55%. Only those who reported that they were working as nurses during the pandemic were included in the analyses, leaving an analytic sample of 1261 nurses for the present study.

When designing the questionnaire, Norway was at top of the first infection wave of the pandemic. However, at the time of the survey (from June 1st to September 30th) the number of COVID-19 patients and deaths in Norway was very low. For instance, the number of hospitalized COVID-19 patients on July 22nd was 3, compared to 325 persons on April 1st, during the peak of the first wave of the pandemic. During the data collection period, most of the intervening measures had been reversed, excluding those related to high-risk activities. The second wave of the pandemic in Norway occurred later in the autumn of 2020, after the survey was closed, and when many of the restrictions were then reintroduced.

### Instruments

#### Demographic

The questionnaire included items on sociodemographic variables such as marital status and caretaker responsibility for children in the household. The questionnaire also included questions about work-related variables such as work schedule (day only, evening only, two-shift including day and evening, night only, three-shift schedule including day, evening and night, and other schedules), type and percentage of full-time position and work place (somatic hospital department, psychiatric hospital department, nursing home, home care service, public health clinic, other work place).

#### COVID-19

The questionnaire included several questions related to the pandemic. “*Have you been infected by the coronavirus?”*, with response alternatives “yes”, “no” and “uncertain”. If they answered yes or uncertain, one follow-up question about severity of symptoms with four response alternatives ranging from “no symptoms” to “severe symptoms” were asked. One follow-up question pertaining to treatment with response alternatives ranging from “no treatment/staying at home” to “treated with respirator” was also asked. Furthermore, all the nurses responded to the question *“Have you been in quarantine due to the coronavirus?”* with response alternatives “yes” or “no”.

#### Sleep

Sleep duration (per 24 h) before the pandemic was measured with one question phrased *“Before the pandemic hit Norway, how long did you usually sleep?”* with response alternatives: “less than 5 hours”, “5–5.9 h”, “6–6.9 h”, “7–7.9 h”, “8–8.9 h”, “9–9.9 h”, “10 hours or more”. One additional question phrased *“After the pandemic hit Norway, how long do you usually sleep?”* with the same response alternatives that were used for sleep duration before the pandemic, was also asked. The responses to the sleep duration question were dichotomized into sleep duration less than 6 h per night and 6 or more hours of sleep per night in line with the recommendations by the National Sleep Foundation [11].

Sleep quality was measured with one question: *“After the pandemic hit Norway, I sleep:”* 1) “much poorer than before”, 2) “to some degree poorer that before”, 3) “no change”, 4) “to some degree better than before” and 5) “much better than before”.

Bedtime, sleep onset time, wake-up time and rise-time were measured with questions with similar phrasing: *“After the pandemic hit Norway”:* “I go to bed … ”, “I fall asleep … ”, “I wake up … ”, “I rise … ” with the response alternatives being 1) “more than one hour earlier than before”, 2) “between 15 and 60 minutes earlier than before”, 3) “as before (less than 15 minutes difference)”, 4) “between 15 and 60 minutes later than before” and 5) “more than one hour later than before”.

### Ethical consideration

The study was approved by the Regional Committee for Medical and Health Research Ethics of Western Norway (REK-West, no 088.08). The study was carried out in accordance with relevant guidelines and regulations, and informed written consent was obtained from all participants included in the study.

### Statistics

IBM SPSS Statistics 27 for Windows was used for the statistical analyses.

Descriptive statistics for all relevant variables were calculated. McNemar chi-square tests were used to compare sleep duration categories before and after the pandemic hit Norway.

## Results

The majority of the nurses were females (90.4%), and mean age at the time of data collection was 44.0 years (SD = 8.0, range 33–69). Of the total sample, 80.2% of the nurses were married/cohabiting and 72.2% had children living at home. Mean number of children was 2.1, ranging from 1 to 6 children.

A total of 32.6% were working day work, 28.6% were working two-shift (day and evening), 7.1% were working night work only, 28.1% were three-shift workers (day, evening and night), and 3.6% were working other schedules involving night work. The majority, 83.8%, worked more than 75% of full time equivalent. Most of the nurses (51.9%) worked in hospital/somatic departments, 11.7% in hospital/psychiatric departments, 10.6% nursing homes, 7.9% in home care services, 6.1% in public health clinics and 11.9% in other work places, respectively.

Of the total sample, 1.2% (*n* = 15) had been infected by the coronavirus, and 4.7% (*n* = 59) of the nurses responded “uncertain” to the question of whether they had been infected. Of the nurses who had had COVID-19, two did not respond to the question about severity, 6 reported mild symptoms, 6 reported moderate symptoms and 2 reported severe symptoms. Of the nurses who were uncertain, 44 did not answer the question about severity, one reported no symptoms, 9 reported mild symptoms, and 5 reported moderate symptoms. Only one of the nurses who had been infected by the coronavirus reported being hospitalized due to the infection. In addition, 27.4% (*n* = 343) of the nurses had been in quarantine due to the pandemic.

The majority of nurses (84.2%) reported no change in sleep duration after the first wave of the COVID-19 pandemic compared to before, 11.9% reported less sleep, and 3.9% reported more sleep. Sleep duration before and after the first wave of the pandemic is presented in Table 1.

**Table 1:** Sleep duration among nurses before and after the first wave of the pandemic in Norway

Sleep duration	Before COVID-19 (*n* = 1257)	After the first wave (*n* = 1258)	*p*-value *
Less than 5 h	0.8% (*n* = 10)	1.7% (*n* = 21)	<.0005
5–5.9 h	10.2% (*n* = 128)	14.2% (*n* = 179)	
6–6.9 h	41.8% (*n* = 526)	41.5% (*n* = 522)	
7–7.9 h	39.6% (*n* = 498)	34.8% (*n* = 438)	
8–8.9 h	7.3% (*n* = 92)	7.3% (n = 92)	
9–9.9 h	0.1% (n = 1)	0.2% (n = 3)	
10 h or more	0.2% (n = 2)	0.2% (*n* = 3)	

^*^McNemar test

There was a significant increase in the dichotomized sleep duration variable (number of nurses sleeping less than 6 h per night), from 11.0% before the pandemic to 15.9% after the pandemic hit Norway (McNemar, *p* < .0005).

A total of 16.2% of the nurses reported much poorer or to some degree poorer sleep quality after the first wave of the COVID-19 pandemic compared to before. At the same time, 1.4% reported much better or to some degree better sleep quality than before the pandemic, and 82.4% reported sleep quality as before.

The results of bedtime, sleep onset time, wake-up time and rise-time are presented in Table 2.

**Table 2:** Descriptive data of bedtimes, sleep onset times, wake-up times and rise-times among Norwegian nurses before and after the first wave of the COVID-19 pandemic

	More than 1 h earlier	15 to 60 min earlier	As before (<than 15 min)	15 to 60 min later	More than 1 h later
Bedtime (n = 1258)	1.7%	4.8%	83.9%	7.4%	2.2%
Sleep onset time (*n* = 1256)	0.6%	2.0%	86.4%	8.8%	2.3%
Wake-up time (*n* = 1254)	2.0%	7.0%	87.0%	3.3%	0.7%
Rise-time (*n* = 1249)	1.0%	3.6%	89.0%	6.0%	0.5%

## Discussion

The purpose of this study was to investigate sleep patterns among Norwegian nurses during the COVID-19 pandemic compared to a retrospective account of how they slept before the pandemic. In particular, the data collection took place from June to September 2020, that is, during a period with low infection rates and a largely reopened society following the first lockdown period. The majority of nurses reported no difference in daily sleep duration or sleep quality during the pandemic compared to before, and bedtime and wake-up times were also as before the pandemic hit Norway. However, 11.9% reported shorter sleep duration and 3.9% reported longer sleep duration during the pandemic. In all, 16.2% of the nurses reported much poorer or to some degree poorer sleep quality than before. Although the majority of nurses reported maintaining a sleep schedule as before the pandemic, 9.6% of the nurses reported going to bed later and 9.0% reported waking up earlier than before the pandemic.

The Norwegian Institute of Public health has defined health care personnel to be at increased risk of being infected by the coronavirus [12]. However, only 1.2% of the nurses reported to have been infected by the virus. This number is in harmony with the overall numbers of infected individuals in the general population of Norway at the same time. The Norwegian Institute of Public Health reported in May 2020 that the first wave of the pandemic in Norway was over. They estimated that nearly 1 % of the population at this time had been infected [13].

Our findings are in contrast to a recent study among nearly 17,000 health care workers from Qatar, in which 10.6% tested positive for COVID-19 between March and June 2020 [14]. Similarly, a study from India conducted between April and August 2020 found a prevalence of COVID-19 of 11% among health care workers [15]. It should be noted that the majority of the participants (74.3%) in the Indian study were frontline health care workers. In the present study we do not have specific information regarding the nurses’ work situation related to the current pandemic, and we do not know the degree to which nurses were exposed to an increased risk of being infected at the workplace. Nevertheless, despite the low self-reported infection rates in this cohort, 27.4% reported being quarantined in the period which could suggest an overall increased risk of being infected. However, Norway has thus far managed to maintain a low infection rate in the population, compared to most other countries [16].

In the present study, 11.9% of the nurses reported shorter sleep duration than before, and at the same time about 9% reported later bedtimes and earlier wake-up times compared to before the pandemic. There was also a significant increase in the proportion of nurses who reported sleeping shorter than 6 h per night, from 11.0% before to 15.9% after the pandemic hit Norway. However, most of the nurses in the present study (84.2%) reported no change in sleep duration related to the pandemic, and the majority reported bedtimes and wake-up times as before, which suggests that the pandemic did not affect most nurses’ sleep – contrary to that indicated by others (e.g., 5). A possible explanation for few significant findings in the present study is that the data collection took place during a period of low infection rates and during a reopening of society after the first lockdown in Norway. Furthermore, Norway has overall had lower infection rates compared to most countries [17] which also may partly explain the absence of negative impact on sleep patterns in the present study.

A meta-analysis by Zeng and colleagues including 53 studies reported a pooled prevalence of poor sleep quality in up to 61% of nurses in general [18]. Furthermore, it is suggested that typical sleep parameters affected by the COVID-19 pandemic could include sleep latency, sleep duration, and sleep efficiency [5]. The findings included in the meta-analysis by Pappa and colleagues conducted in April 2020, reported a pooled prevalence of insomnia of 34.3% among health care workers [8]. In addition, one survey from China by Zhang and colleagues showed that more than one-third of all medical staff experienced symptoms of insomnia associated with the COVID-19 pandemic [3]. The majority of nurses in the present study, however, reported no change in sleep duration or quality after the pandemic hit Norway, which suggests that the pandemic had limited impact on sleep. As discussed in earlier, this may be related to the timing of the data collection in the present study. Still, the present study did not include instruments measuring sleep problems and insomnia, like the Pittsburgh Sleep Quality Index [19], ithe Insomnia Severity Index (ISI) [20] or the Bergen Insomnia Scale (BIS) [21], which makes it difficult to compare with the studies that have used such assessments. However, 16.2% of the nurses in our study reported much poorer or to some degree poorer sleep quality as compared to before the pandemic, which implies that some of the nurses did experience deterioration of sleep co-occurring with the pandemic. Based on studies from China, performed during the peak of the first wave of the pandemic, reporting high prevalence of sleep problems [2, 4] one could speculate that the prevalence of sleep problems would have been higher if the data collection took part during the peak of a pandemic wave, instead of between peaks. The fact that more than 16% of the nurses reported problems and nearly 12% of the nurses slept shorter than before may indicate that sleep problems persisted during this calm period and may have become chronic in these nurses.

## Conclusion

Most existing literature that has examined sleep among health care workers during the COVID-19 pandemic has been carried out during periods of high infection rates. The present study investigated sleep patterns among Norwegian nurses following the first wave of the pandemic – during a period with low rates of COVID-19. The majority of the nurses reported no difference in sleep duration, sleep quality, and bedtime and wake-up times during the pandemic compared to before. Still, nearly 12% reported shorter sleep duration, and about 16% reported poorer sleep quality than before, indicating that some nurses experienced worsening of their sleep also in this calm period of the pandemic. This could also suggest that the sleep problems are of such severity that they persist in the period after the peak of the pandemic wave. However, a worsening of sleep could also be related to other factors than the pandemic itself. The majority of nurses went to bed and woke up as before, but around 9% of the nurses reported later bedtimes and earlier wake-up times than before the pandemic. Sleep problems related to the COVID-19 pandemic may become chronic and long-lasting. It is therefore important to investigate how sleep patterns vary throughout and after the pandemic.

### Strengths and limitations

Firstly, this cohort included a relatively large sample size comprising a homogenous group of mostly female nurses, which reduces the impact of confounders on the results, and is thus a strength of the present study. However, this also represents a limitation with the study in terms of the generalizability of the findings, especially when it comes to males and other occupational groups. The cross-sectional nature of the study does not allow interpretation about causality. Thirdly, we did not assess if study participants were frontline or non-frontline nurses, which is a factor that is likely to affect the nurses sleep during this COVID-19 pandemic. It is also a limitation that sleep was measured with questionnaires rather than using objective recordings, although sleep quality is first and foremost a subjective matter. The measurement of sleep was also conducted using single item questions not previously validated, which makes the validity of the measures somewhat unclear. There is also uncertainty associated with the questions that required participants to retrospectively report on their sleep before the pandemic. For instance, it cannot be ruled out that remembering the past as being more positive than it actually was may have led to an expectancy that COVID-19 related changes are more negative. Hence, the current estimates may be affected by recall bias. Hence, these estimates may be affected by recall bias. A further limitation of the study is that the sleep questions related to the pandemic did not explicitly instruct the participant to think of the period with low infection rates, but instead asked more generically about sleep during the pandemic. This makes it unclear whether the results of this study actually reflect the sleep pattern during a period of low infection rates (i.e. at the time of the assessment), or whether they reflect sleep more generally during the pandemic.

## Data Availability

The datasets used and/or analysed during the current study available from the corresponding author on reasonable request.

## Abbreviations

COVID-19: COrona VIrus Disease-2019
SUSSH: SUrvey of Shift work, Sleep and Health
WHO: World Health Organization
PSQI: Pittsburgh Sleep Quality Index
ISI: Insomnia Severity Index
BIS: Bergen Insomnia Scale
